# Assessment of Prenatal Exposure to Arsenic in Tenerife Island

**DOI:** 10.1371/journal.pone.0050463

**Published:** 2012-11-28

**Authors:** Oriol Vall, Mario Gómez-Culebras, Oscar Garcia-Algar, Xavier Joya, Dinoraz Velez, Eva Rodríguez-Carrasco, Carme Puig

**Affiliations:** 1 Unitat de Recerca Infància i Entorn (URIE), Institut Hospital del Mar d’Investigacions Mèdiques (IMIM), Barcelona, Spain; 2 Red Samid, Retic, Instituto de Salud Carlos III, Madrid, Spain; 3 Servei de Pediatria, Hospital del Mar, Barcelona, Spain; 4 Universitat Autònoma de Barcelona, Barcelona, Spain; 5 Departamento de Cirugía Pediátrica, Universidad de Tenerife, Tenerife, Spain; 6 Instituto de Agroquímica y Tecnología de Alimentos, Consejo Superior de Investigaciones Científicas (IATA-CSIC), Valencia, Spain; Public Health Agency of Barcelona, Spain

## Abstract

**Introduction:**

Increasing awareness of the potential chronic health effects of arsenic (As) at low exposure levels has motivated efforts to better understand impaired child development during pregnancy by biomarkers of exposure. The aims of this study were to evaluate the prenatal exposure to As by analysis of an alternative matrix (meconium), to examine its effects on neonatal outcomes and investigate the association with maternal lifestyle and dietary habits during pregnancy.

**Methods:**

A transversal descriptive study was conducted in Tenerife (Spain). A total of 96 mother-child pairs participated in the study. A questionnaire on sociodemographic, lifestyle and dietary habits during pregnancy was administered the day after the delivery. Analysis of total As in meconium was performed by inductively coupled plasma-optical emission spectrometer.

**Results:**

Total As was detected in 37 (38.5%) meconium samples. The univariate logistic regression model indicates that prenatal exposure to As was associated with a low intake of eggs per week (OR 0.56; CI (95%): 0.34–0.94) during pregnancy. Conversely, frequent intake of vegetables was associated with prenatal As exposure (OR: 1.19; CI (95%): 1.01–1.41) and frequent intake of processed meat (as bacon, Frankfurt’s sausage, and hamburger) shows a trend to As prenatal exposure (OR: 8.54; CI (95%): 0.80–90.89). The adjusted multivariate logistic regression model indicates that only frequent intake of vegetables maintains the association (OR: 1.31; CI (95%): 1.02–1.68).

**Conclusion:**

The studied population presented a low As exposure and was not associated with neonatal effects. Maternal consumption of vegetables during pregnancy was associated with detectable meconium As levels; however the concentration detected in meconium was too low to be considered a major public health concern in this geographical area.

## Introduction

The inorganic form of arsenic present in drinking water is considered the most toxic form and classified as a human carcinogen by the International Agency for Research on Cancer [1]. Arsenic in food is mainly present in the organic form, basically as methylated metabolites that are easily eliminated from body and widely assumed to be less toxic.

Arsenic is a metalloid that is found in the environment from natural sources (e.g. volcanic activity and weathering of minerals) and from anthropogenic activity (e.g. industry and agriculture). Humans are mainly exposed to arsenic via the environment while in a small group of the population it can be also an occupational hazard. The general population could be exposed to arsenic mainly through water and food. In some regions of Bangladesh, China, Chile and United States of America drinking water is the major source of human exposure due to the fact that ground water contains arsenic at concentrations in excess of 50 µg/L. Among the dietary source arsenic can be found mainly in seafood, rice cereal and poultry [2].

Toxicological data is derived mainly from studies on arsenicosis: drinking inorganic arsenic-rich water over long periods of time which occurs usually in areas with major public health concerns. Study results indicate various health effects in adults including cancer, skin lesions, cardiovascular diseases and diabetes [3]–[6]. Adverse outcomes of pregnancy such as increased risk of spontaneous abortion, stillbirth, preterm birth and neonatal death at elevated water arsenic concentrations have also been reported [7], [8].

Some studies described that both, inorganic arsenic and the methylated metabolites can easily cross the placenta [9]. There is emerging evidence that early-life exposures affect foetal and infant growth, mainly by epigenetic effects [10]. The developing organism is particularly vulnerable to toxic insults, because of rapid cell division and differentiation, especially in the nervous system. Subsequently an increased awareness of the potential chronic health effects of arsenic at low exposure levels has motivated efforts to better understand morbidity and mortality, as well as impaired child development during pregnancy by using objective biomarkers of exposure [11].

Meconium is a matrix that has been used to detect foetal exposure over the last 6 months of pregnancy to different xenobiotics. To date, there is only one published study on the foetal exposure to arsenic and other heavy metals with this matrix [12].

Therefore, the aims of this study were to evaluate the prenatal exposure to arsenic, examine the effects on neonatal outcomes and investigate the association with maternal lifestyle and dietary habits during pregnancy.

## Materials and Methods

### Population and Study Design

A cross sectional descriptive pilot study was conducted in Tenerife, the largest and most populated island of the seven islands that make up the archipelago of the Canary Islands (Spain). The economy of Tenerife is based on a few sectors: tourism (78%) industrial activities (9%) and, to a lesser extent, farming and fishing (2%). Agriculture is centred on the northern slopes. In the last decades, farming in these Islands has turned to an intensive kind of agriculture (plastic greenhouses). Although there is a diversity of industrial activities on the island, the most important are the oil refineries in the capital Santa Cruz de Tenerife. There is no published data on environmental As values in water, air or ground.

The study was conducted in the Paediatric Department of the Hospital La Candelaria, the biggest hospital in Tenerife. A total of 96 pregnant women agreed to participate in the study and signed an informed consent form. Enrolment period was between August 2006 and June 2007. The study was approved by the local Ethics Committee (Comité de Ética e Investigación Clínica (CEIC), Hospital la Candelaria, Santa Cruz de Tenerife) in accordance with the Helsinki Declaration.

### Analytical Procedure for Arsenic Assessment

Neonatal meconium was collected from the infant’s diaper. The sample was aliquoted (1g meconium) and kept frozen at −20°C until analysis.

Total arsenic in meconium was quantified with minor modifications following the procedure reported previously by Farias et al [13]. All chemicals were of analytical reagent grade unless otherwise stated. Deionized distilled water (DDW) was used for the preparation of reagents and standards. A commercially available solution of As (V) (1000 mg/L) (Merck, Darmstadt, Germany) was used to prepare standard working solutions. Nitric acid (Merck) was additionally purified by distillation in a quartz still. H_2_O_2_ and HClO_4_ were supplied by Merck. Analysis of total arsenic in the meconium was performed by using an inductively coupled plasma-optical emission spectrometer (ICP–OES) (Optima 3100 XL, axial view; Perkin Elmer, Norwalk CT, USA) equipped with an autosampler (model AS-90, Perkin Elmer) after a microwave-assisted acid digestion. Calibration curves were obtained with standards prepared in 0.7 M HNO_3_. Despite the complexity of the matrix investigated, calibration by the standard addition procedure was not necessary. The detection limit of the assay was 0.33 microg g(-1)dw (dry weight). The concentration of As in meconium is expressed in ng/ml (ppb). Recalculation of the concentrations in nanogram of As per gram of meconium can be done since the amount (g) of meconium and the solvent volume used to resuspend meconium are known.

### Study Variables

A structured questionnaire on sociodemographic, lifestyle and dietary habits during pregnancy was administered to all participants the day after the delivery. Maternal obstetric history, neonatal somatometry and clinical examination at birth were also recorded.

Sociodemographic questionnaire asked for parental education, working status, social class and country of origin, area of residence and maternal age. Socioeconomic status was defined by a widely used Spanish adaptation of the British classification system [14].

Variables regarding lifestyle during pregnancy included smoking and exposure to environmental tobacco smoke, alcohol, drugs of abuse and medicinal products consumption. Moreover, chemical exposure at work or at home was recorded.

The dietary information about pregnancy period was collected using a food frequency questionnaire of 16 items. Participants were asked how many times per week, on average, they had consumed each item during pregnancy. Also, they were asked about the origin of their drinking water.

### Statistical Analysis

Descriptive statistics of As skewed distribution in meconium were performed using mean, median, geometric mean and percentiles. The dependent variable was a dichotomous arsenic level (detected vs non-detected). Spearman’s correlations between the average use of the studied food groups and arsenic detected in meconium were examined. Preliminary association between sociodemographic, life style characteristics of pregnant women and neonatal outcomes with foetal exposure to As were done by Student's t test for continuous variables and Chi-square test for dichotomous variables or multinominal logistic regression for categorical variables. Statistical significance was set at p<0.05. Variables associated significantly with foetal exposure to As were entered into a univariate logistic regression model to determine the independent effects of the covariates on the dependent variable. A multivariate logistic regression model was constructed using an enter mode. Variables that were found to be significant in the likelihood ratio test at a level of p<0.10 were retained in the model and removed at p>0.20. Database management and statistical analysis were performed with SPSS v 14.0 (SPSS, Chicago, IL, USA).

## Results

Total arsenic was detected in 37 meconium samples (38.5%) with a range of 0.10 to 31.40 ng/g of meconium. The mean (SD) and geometric mean concentration of total As were 6.79 (1.05) ng/g and 4.55 ng/g, respectively. Percentiles were determined at 5^th^: 0.40 ng/g, 25^th^: 3.0 ng/g, 50^th^: 5.60 ng/g, 75^th^: 7.4 ng/g and 95^th^: 25.50 ng/g. [Range: 0.01 to 2.49 ng/ml (ppb); Mean (SD): 1.05 (0.13) ng/ml (ppb); Geometric mean 0.62 ng/ml (ppb); Percentiles at 5^th^: 0.03, 25^th^: 0.28, 50^th^: 0.76, 75^th^: 1.77 and 95^th^: 2.30 ng/ml (ppb)].

Tenerife Island is divided into 3 areas; metropolitan, north and south areas. In the south area the meconium samples positive for arsenic were less frequent (detected As 27.6% vs non-detected As 43.5%) than in the metropolitan area (51.7% vs 45.7%) or the north area (20.7% vs 10.9%). The differences between the areas were not statistically significant (p = 0.285). Furthermore, Santa Cruz de Tenerife city, the main municipality in the metropolitan area, has 5 districts. The majority of samples with arsenic detected were in Centro-Ifara district with the old quarter and Port of Santa Cruz (detected As 45.5% vs non-detected As 5.9%),. The frequency of positive meconium for As was lower in the following 3 districts which are mainly residential areas: Ofra-Costa Sur (detected As 9.1% vs non-detected As 5.9%), Salud-La Salle (18.2% vs 41.2%) and Sudoeste (27.3% vs 47.1%). The differences observed did not reach statistical significance (p = 0.081).

Finally, there were no meconium samples collected from Anaga district, a rural and mountainous profile area.

The distribution of parental sociodemographic characteristics in the two categories: As detected and As non detected is shown in Table 1. More than 85% of mothers were Spanish. In the category of newborns with detectable arsenic levels, 34.6% were offsprings of mothers with advanced university degree compared to 4.3% in the category of newborns without detectable arsenic levels in meconium (OR: 12.0; CI (95%): 2.16–66.55); secondary education was taken as reference category. In addition, fewer newborns have been exposed to arsenic when the father’s social class was managerial or technical (15.4% vs 31.0%; OR: 0.09; CI (95%): 0.01–0.61) and partly skilled (30.8% vs 47.6%; OR: 0.11; CI (95%): 0.02–0.67); skilled category was taken as the reference.

**Table 1:** Parental socio-demographics characteristics by prenatal exposure to total arsenic detected in meconium.

			n	AsDetected	n	AsNon-Detected	p-value
**Maternal age** (years), mean (SD)			28	30.9 (4.5)	46	29.9 (5.2)	0.374
**Parental country of origin** (Spain/Other), (%)							
Non-Spanish Mothers			4	14.8	5	10.6	0.597
Non-Spanish Fathers			5	19.2	5	10.6	0.307
**Parental Educational Level** (%)							
** Mother’s**		Primary education & Illiterate	4	15.4	12	26.1	0.057
		Secondary education	9	34.6	24	52.2	
		University degree	4	15.4	8	17.4	
		Advanced University degree	9	34.6	2	4.3	
** Father’s**		Primary education & Illiterate	6	24.0	18	38.3	0.564
	Secondary education		9	36.0	17	36.2	
	University degree		4	16.0	6	12.8	
	Advanced University degree		6	24.0	6	12.8	
**Parental Employment** (yes/no), (%)							
Unemployed Mother			4	15.4	9	19.1	0.687
Unemployed Father			1	3.7	4	8.5	0.428
**Parental socioeconomic status** (%)							
** Mother’s**		Professional	1	4.5	3	7.9	0.403
		Managerial/Technical	8	36.4	6	15.8	
		Skilled	5	22.7	9	23.7	
		Partly skilled	7	31.8	15	39.5	
		Unskilled	1	4.5	5	13.2	
** Father’s**		Professional	5	19.2	5	11.9	0.058
		Managerial/Technical	4	15.4	13	31.0	
		Skilled	7	26.9	2	4.8	
		Partly skilled	8	30.8	20	47.6	
		Unskilled	2	7.7	2	4.8	
**Area of residence** (%)							
Rural (<10.000 hab)			4	13.8	7	15.2	0.888
Semiurban (10–100.000 hab)			12	41.4	21	45.7	
Urban (>100.000 hab)			13	44.8	18	39.1	

Chi-square test; p<0.05.

Children with detectable levels of arsenic had a significantly higher birth weight compared to the children without detectable As (OR: 1.00; CI (95%): 1.00–1.02). No other obstetric and perinatal outcomes were significantly associated with arsenic in meconium (Table 2).

**Table 2:** Obstetric and anthropometric characteristics of the newborns according to the results obtained.

	n	AsDetected	n	AsNon-Detected	p-value
**Previous pregnancies** (%)					
0	16	57.1	28	59.6	0.635
1	10	35.7	13	27.7	
>2	2	7.1	6	12.8	
**Previous premature infants** (yes/no), (%)					
Yes	0	0	0	0	NA
**Previous abortions** (%)					
0	24	85.7	33	70.2	0.282
1	4	14.3	13	27.7	
>2	0	0	1	2.1	
**Children characteristics at birth**					
Gender (male), (%)	17	58.6	21	44.7	0.238
Gestational age (weeks), mean (SD)	27	39.2 (1.3)	45	39.1 (1.2)	0.813
Prematurity (yes), (%)	1	3.7	1	2.2	0.711
Weight (g), mean (SD)	29	3459.3 (537.4)	47	3235.5 (405.2)	0.043
Length (cm), mean (SD)	29	51.2 (2.4)	47	50.7 (2.0)	0.282
Cranial perimeter (cm), mean (SD)	27	34.5 (1.6)	42	34.1 (1.3)	0.196
**Clinical diagnostic at birth** (yes), (%)	11	37.9	13	27.7	0.349
Chromosomal abnormalities (yes), (%)	0	0	2	4.2	0.265
Lost of foetal well-being (yes), (%)Risk of perinatal infection (yes), (%)	1	3.4	0	0	0.195
	6	20.7	6	12.5	0.337
Neonatal hypoglycaemia (yes), (%)	1	3.4	3	6.3	0.591
Neonatal jaundice (yes), (%)	0	0	0	0	NP
Hip dysplasia (yes), (%)	1	3.4	2	4.2	0.875
Other outcomes (yes), (%)	2	6.9	0	0	0.065

NA: Not applicable.

Chi-square test; p<0.05.

Maternal lifestyle, chemical exposure and dietary habits during pregnancy are presented in Table 3. Newborns with As in meconium were less exposed to tobacco smoke, due to the fact that no mother in that category smoked during pregnancy and fewer mothers were exposed to second hand smoke (OR: 0.16; CI (95%): 0.03–0.80).

**Table 3:** Maternal lifestyle, chemical exposure and dietary habits during pregnancy.

	n	Detected As	n	Non-DetectedAs	p-value
**Maternal tobacco smoke exposure** (yes), (%)					
Smoke before pregnancy	4	17.4	12	29.3	0.292
Smoke during pregnancy	0	0	8	17.0	0.021
Exposed to tobacco smoke during pregnancy	2	9.5	15	39.5	0.015
Father smoking during pregnancy	4	14.8	17	37.0	0.044
**Drug abuse** (yes), (%)	0	0	0	0	NA
**Alcohol consumption** (yes), (%)	0	0	1	2.1	0.429
**Medicine use** (yes), (%)	25	86,2	40	85,1	0.895
Antidepressants	2	7.4	0	0	0.059
Vitamin supplements	8	30.8	5	11.4	0.044
Antibiotics	0	0	8	18.2	0.021
**Chemical exposure** (yes), (%)					
At work	4	13.8	6	12.5	0.870
At hobby	3	10.3	0	0	0.026
Live near pollutant areas	4	14.3	6	12.8	0.851
Use of pesticides in the yard	5	18.5	4	11.4	0.432
Use of head lice treatment	1	3.6	2	4.3	0.870
Use of mothballs	0	0	3	6.5	0.168
Use of products to control fleas on your pet	9	32.1	18	39.1	0.545
**Water source** (%)					
Private well	1	3.3	1	2.0	0.582
Running water	4	13.3	11	22.4	
Mineral water	25	83.3	37	75.5	
**Diet** (times per week), mean (SD)					
Full-Fat Milk	11	14,5 (6.1)	20	13,7 (6.1)	0.714
Fat-Free Milk	17	14,3 (10.0)	29	14,1 (6.3)	0.906
Fruit	28	9,5 (6.9)	47	7,6 (4.5)	0.140
Vegetables	28	6,5 (3.3)	47	4,9 (2.8)	0.031
Eggs	28	2,1 (1.0)	46	3,0 (2.0)	0.039
Butter and/or Margarine	16	4,1 (2.4)	26	3,6 (2.5)	0.541
Legume and Rice	27	1,9 (1.6)	42	1,7 (1.0)	0.452
Nuts	10	2,3 (1.8)	13	1,9 (1.2)	0.564
Red Meat	24	1,8 (1.7)	43	1,9 (0.8)	0.812
Processed Meat (bacon, hamburguer, Frankfurt sausage)	4	2,0 (0.8)	12	1,2 (0.4)	0.033
White Meat	26	1,8 (0.8)	46	2,1 (0.8)	0.169
Fish	27	1,9 (0.8)	36	1,8 (0.7)	0.587
Precooked Food	8	2,1 (1.3)	14	1,6 (1.1)	0.306
Canned Food	24	2,0 (0.9)	36	2,0 (1.5)	0.935
Commercial Juice	16	6,2 (3.2)	38	6,4 (4.9)	0.877
Soft Drinks	10	3,3 (2.3)	21	5,2 (4.3)	0.198

NA: Not applicable.

Chi-square test; p<0.05.

The source of mother's water for consumption does not appear to be a determining factor of arsenic exposure. Conversely, a frequent intake of vegetables was associated with prenatal exposure to arsenic (OR: 1.19; CI (95%): 1.01–1.41). A frequent intake of processed meat (as bacon, Frankfurt’s sausage, and hamburger) was associated with As in meconium but that association did not reach statistical significance (CI (95%): 0.80–90.89). On the other hand, low weekly intake of eggs was associated with prenatal exposure to arsenic (OR: 0.56; CI (95%): 0.34–0.94).

Table 4 shows the OR and 95%CI obtained in the unadjusted and adjusted logistic regression model. This model includes only the statistically significant variables from the univariate logistic regression model.

**Table 4:** Multivariate regression model.

	OR	CI (95%)	OR Adj	CI (95%)
Exposed to tobacco smoke during pregnancy	0.30	0.03–1.36	0.27	0.01–1.32
Vegetables	1.13	1.00–1.54	1.17	0.99–1.75
Eggs	0.77	0.21–1.23	0.64	0.18–1.09

Adj: Adjusted for maternal age, newborn sex, birth weight.

Chi-square test; p<0.05.

Maternal average intake of vegetables during pregnancy was the single factor significantly and independently associated with prenatal exposure to As by meconium analysis in our study.

Arsenic concentration in meconium was significantly correlated only with average consumption of white meat (Spearman’s rho: −0.45; p = 0.02). However, it was not significantly correlated with average consumption of vegetables (Spearman’s rho: 0.02; p = 0.90), eggs (Spearman’s rho: −0.22; p = 0.25) and processed meat (Spearman’s rho: 0.63; p = 0.37).

## Discussion

In our study, 38.5% of the newborns had detectable arsenic in meconium. Our population has a low arsenic exposure and these arsenic levels were not associated with obstetric and neonatal effects. Maternal consumption of vegetables during pregnancy was the only factor associated with detectable meconium arsenic levels.

A limitation of our study was that we do not have data on arsenic speciation in order to differentiate between inorganic and organic arsenic exposure. Consequently, a risk assessment not taking into account the different species but considering total arsenic as being present exclusively as inorganic arsenic would lead to a considerable overestimation of the health risk related to dietary arsenic exposure. However, exposure levels in our population are below the normal levels in different biological matrixes. Another limitation is the poor correlation between As detected and median value of the average consumption of foods compared with other studies, probably because it was expressed as frequency of use (times per week) and not as µg/day/person. Finally, our results are difficult to generalize since this study was undertaken only in Tenerife and the sample size was small.

### Prenatal Exposure to As by Meconium Analysis

Arsenic levels in blood, urine, hair and nails have all been investigated and used as biological indicators of exposure to arsenic. In human blood, typical values in non-exposed individuals are in the range of 0.5 to 2 µg/L. However, the urine is generally accepted as the most reliable indicator of recent arsenic exposure. The normal level for an unexposed person is usually less than 50 µg/L [15]. Maternal exposure to the environmental arsenic during pregnancy has been demonstrated in several articles. The median (interquartile range) concentration of urinary arsenic detected at 30 weeks was 84 (42–230) µg/L in pregnant women from Bangladesh [16] and maternal blood 5.30 (3.39–8.55) µg/L and cord blood 3.71 (2.50–6.41) µg/L in an epidemiological study from China [17]. Furthermore, arsenic tends to accumulate in hair and nails, and measurement of arsenic levels in these tissues may be a useful indicator of past exposures. Normal levels in hair and nails are 1 µg/g (ppm) or less [18].

Meconium is a matrix that has been used to detect foetal exposure to different xenobiotics due to the fact that it can be obtained easily, the collection is not invasive, is a repository of these substances and their metabolites and is representative of a wide period of exposure starting at 12^th^ week of pregnancy. However, the only study published that evaluates the prenatal exposure to heavy metals did not detect arsenic in this matrix [12]. The present study is the first one published that has detected total As in 38.5% of meconium analysed. Only 19 meconium samples (51.35%) had As concentrations above 1 ng/ml (ppb) and no sample had As concentration higher than 1 µg/ml (ppm). Therefore, the arsenic levels detected in the meconium of newborns in our study were far below the safe threshold mentioned above for hair and nails as biological matrices of chronic or past exposure.

Recently, prenatal exposure to As was estimated by testing it in placenta [19]. A placental arsenic concentration of 34 µg/g was reported in a region of Argentina with high concentrations of arsenic in drinking water. Placental arsenic concentrations of 7 and 23 µg/g were reported in a control and smelter area from Bulgaria, respectively [20]. Due to the lack of studies regarding As exposure estimated by meconium testing there are no references for the purpose of comparing with our results. As an assumption, we can use levels detected in placenta, as a foetal tissue that may be a useful indicator of prenatal exposure. In our study the maximum As determined in meconium was 31.40 ng/g, approximately 1000 fold lower than found in placenta.

The analysis of total As in meconium was performed by ICP-OES even though atomic absorption spectrometry is usually the method of choice for assessing exposure to arsenic. The low concentration of some trace elements in the biological samples demands a technique with high sensitivity. In this sense, ICP-OES has lower detection and quantification limits than atomic absorption spectrometry. However, the small amount of meconium collected did not allow for arsenic speciation in meconium.

### Effects of Prenatal As Exposure on Obstetric and Neonatal Outcomes

Increased risk of spontaneous abortion, stillbirth, preterm birth and neonatal death at elevated water arsenic concentrations were indicated by some authors, when mothers were interviewed about previous pregnancies [21]. Moreover, other authors point out a moderately increased risk of impaired foetal growth and increased foetal and infant mortality with prenatal exposure to arsenic [22]–[24]. There are also few studies indicating that infants born to women who drink water with elevated arsenic concentrations during pregnancy have a lower birth weight [25]–[27]. However, the arsenic levels to which newborns were exposed in our study did not show any appreciable harm neither with respect to maternal obstetric history nor to neonatal outcomes, with the exception of the birth weight. In our study, birth weight of newborns exposed to As prenatally was significantly higher than of the non-exposed infants. There is no obvious biological mechanism by which arsenic could increase birth weight. Arsenic has no apparent beneficial health effects for humans. However, it is known that low levels of several organic arsenic containing chemicals are used as growth promoters for poultry and swine [28].

### Maternal Lifestyle and Food Dietary Habit during Pregnancy

Considering maternal lifestyle, some authors pointed out that tobacco may also be a route of exposure of As, mainly for smokers, because tobacco crops could be treated with arsenate sprays although it is likely that cigarettes sold today do not contribute more than 0.01 to 0.1 µg of As per cigarette in mainstream smoke. Results in our study suggest that exposure to tobacco smoke is not an important source of As. Newborns with detectable meconium arsenic levels showed to be less exposed to tobacco smoke during pregnancy in accordance to maternal self-reporting.

Moreover, it is known that arsenic containing substances are used in medicine as antiparasitic and genitourinary antibiotics. Some traditional remedies in Chinese Medicine and Ayurvedic Medicine can include arsenic-containing minerals. As well, it can be detected in cosmetic products and in tattoos and other permanents pigments. Our study highlighted a possible source of exposure given that newborns exposed to arsenic are associated with mothers who declared to take vitamin supplements during pregnancy.

Arsenic is present at different levels in water depending on its source. The current World Health Organisation [29] guideline and the Environmental Protection Agency (EPA) from USA have decreased the limit in drinking water from 50 to 10 µg/L [29], [30]. The maximum allowed As level in drinking water according to the Spanish laws is also 10 µg/L. Our results suggest that drinking water in Tenerife seems not to be a risk factor.

In 2009, the European Food Safety Authority Panel on Contaminants in the Food Chain assessed the risks to human health related to the presence of As in food. The highest total arsenic levels were measured in the following food commodities: fish, seafood and rice. More recently, methods for determination of inorganic arsenic have become available and showed that apart from drinking water, which is well known to have a great importance, rice and seafood products could also contribute to inorganic arsenic exposure in low amounts [31]. A more relevant estimate is based on the assumption that a maximum of 5% of the arsenic ingested via seafood is inorganic [32]. Similar to other countries, in Spain, the main contributor to total dietary As intake is seafood, whereas rice is the main contributor for inorganic As. The estimated dietary intake of As ranges between 354 and 360 µg/day/person. Compared to total As daily mean intakes in other countries, the figures in Spain are in the higher range of the reported values, similar to Japan [33].

In our study, the contribution of each broad food category to total arsenic exposure was calculated from the median value expressed in times consumed per week during pregnancy as declared by the mother. Our results show that one of the contributors to total overall arsenic exposure were the vegetable products. The Canary Islands, rugged and volcanic, are not considered having an arsenic-rich soil and it is accepted that As is present only as residual due to environmental contamination. In 2006 and 2007, arsenic concentrations in air ranged from 0.19 and 0.23 ng/m^3^, respectively, and did not exceed the acceptable levels (6 ng/m^3^) according to the Spanish law (RD102/2011). Probably, the vegetables originated from plants cultivated in soils with higher arsenic content or were irrigated with water containing arsenic as found by Moyano et al in an agricultural area of central Spain [34]. Another explanation could be that substantial amounts of organic arsenic containing pesticides were used as indicated since 1990.

### Conclusions

The determination of toxic elements in the biological samples of human beings is an important clinical screening procedure. In our study total arsenic in meconium was detected. In our population, with low exposure, arsenic levels were not associated with neonatal effects. Maternal consumption of vegetables during pregnancy was the only factor associated with detectable meconium arsenic levels. The concentrations found in meconium were too low to be considered a major public health concern in the geographical area were the study was conducted.

Elemental determination in human tissues is the most common application of biological monitoring for screening, diagnosis and assessment of metals and metalloid exposures and their risks. Therefore, further epidemiologic studies are required to confirm the results suggested and for understanding the mechanisms related with prenatal As exposure.
